# Vision-Based Cable Displacement Measurement Using Side View Video

**DOI:** 10.3390/s22030962

**Published:** 2022-01-26

**Authors:** Geonhee Lee, Sunjoong Kim, Sangsub Ahn, Ho-Kyung Kim, Hyungchul Yoon

**Affiliations:** 1 Department of Disaster Prevention Engineering, College of Engineering, Chungbuk National University, Cheongju 28644, Korea; korrjdgml@daum.net; 2 Department of Civil Engineering, College of Engineering, University of Seoul, Seoul 02504, Korea; sunjoong@uos.ac.kr; 3 Korea Expressway Corp., Hwaseong 18489, Korea; ahnss@ex.co.kr; 4 Department of Civil and Environmental Engineering, College of Engineering, Seoul National University, Seoul 08826, Korea; hokyungk@snu.ac.kr; 5 School of Civil Engineering, College of Engineering, Chungbuk National University, Cheongju 28644, Korea

**Keywords:** vision-based displacement measurement, cable-stayed bridge, side view video, computer vision

## Abstract

Recent tragedies around the world have shown how accidents in the cable-stayed bridges can wreak havoc on the society. To ensure the safety of the cable-stayed bridges, several studies have estimated the cable tension force using the vibration of cables. Most of these methods for estimating the tension of a cable start with measuring the displacement of the cable. Recent development of commercial cameras provide opportunity for more convenient and efficient method for measuring the displacement of cable. However, traditional vision-based displacement measurement methods require the assumption that the movement of the cable should be measured in parallel to the camera plane. This assumption limits the installation location of the camera when measuring the displacement of a cable. Therefore, this study introduces a new vision-based cable displacement measurement system that can measure the displacement of a cable in various locations even when the camera is installed in the side of the cable. The proposed method consists of three phases: (1) camera projection matrix estimation, (2) cable tracking in the image coordinate, and (3) cable displacement estimation in the world coordinate. To validate the performance of the proposed method, a simulation-based validation test, a lab-scale validation test, and an on-site validation test were conducted. The simulation-based validation test verified the performance of the proposed method in an ideal condition, and the lab-scale validation test showed the performance of the method in physical environment. Finally, the on-site validation test showed that the proposed method can measure the cable displacement with a side view camera.

## 1. Introduction

Recent tragedies around the world showed how accidents in the cable-stayed bridges can wreak havoc on the society. In 2015, a lightning accident at the Seohae Grand Bridge in Pyeongtaek, Gyeonggi-do, South Korea caused damage and breakage of cables, resulting in casualties [1]. In addition, at the Cheonsadaegyo Bridge in Mokpo, Jeollanam-do, South Korea, which was completed in 2019, citizens are feeling anxious due to the vibration of the bridge cables [2]. Furthermore, in Minneapolis, Minnesota, USA in 2012, large and small damages are occurring due to cables, such as cable breaking and the closing of the pedestrian bridge [3].

Various studies have been conducted to monitor the condition of the cable-stayed bridge by estimating the cable tension force. Kim et al. estimated cable tension forces based on the natural frequency of cables [4], and Yin et al. analyzed the cable tension force using the response of a vehicle passing on a cable-stayed bridge [5]. Cho et al. measured the cable tension force using three different methods [6], and Yim at al. estimated the cable tension force of cable-stayed bridge cables using a stress sensor with the elasto-magnetic (EM) effect of ferromagnetic materials [7]. Zhao et al. also used microwave intrametric radar to measure the displacement of the Nanjing Eye cable-stayed bridge and estimate the tension force [8]. Most of these studies required the measurement of cable displacement either directly or indirectly.

The displacement of a structural component can be measured in different methods. The most direct way to measure displacement is using a linear variable differential transducer (LVDT), but requires additional scaffolds to be installed to the structure [9]. Another method for measuring displacement is to measure the acceleration and double integrating the acceleration to obtain the displacement [10,11,12]. Installing an accelerometer is relatively convenient compared to using a LVDT, but have large accumulative errors in the integration process. Hong et al. improved the accuracy of the displacement by using the Tikhonov regularization technique, and Kandula et al. used an adaptive block signal model with automatic sequence detection to improve the accuracy of the displacement, but the displacement in the low frequency ranges was still unreliable [13,14]. In addition, a method for using a global positioning system (GPS) to measure a displacement in a bridge girder was introduced, but the low-cost GPS receiver could not provide the sufficient accuracy for structural monitoring purpose [15].

A vision-based displacement measurement system is relatively economical compared to the conventional method. Lee et al. measured the vibration of the bridge by recording video of a target installed on the top of the cable-stayed bridge [16]. Ribeiro et al. installed a target on the lower part of the railway bridge to measure the displacement, and Lee evaluated the possibility of vision-based displacement measurement technology with a cable-stayed bridge model [17,18]. Furthermore, Yoon et al. introduced a target-free vision-based displacement measurement method, and introduced a method to measure the displacement of a structure using an unmanned aerial vehicle [19,20,21]. Tian et al. developed a method to measure the displacement by using the LSD (Line Segments Detector) algorithm without a target by photographing the cables of the suspension bridge [22].

Most of the vision-based structure displacement measurement techniques lies on the assumption that the motion of the object is parallel to the image plane. Therefore, the camera must be installed perpendicular to plane of the structural motion. However, it may be difficult to find the camera location that satisfies these conditions in cable stayed bridges due to the traffic which can block the line of sight. The camera should be relocated to the side of the cable to secure the line of sight, but the measured displacement using the video recorded from the side view will contain a large projection error.

Therefore, this paper introduces a new method that can measure the displacement even when a camera is installed with a side angle view. The proposed system tracks the feature points of the side view video, and calibrates the projection error induced by the side angle using the camera projection matrix. The system is comprised of three phases: estimating the camera projection matrix, tracking the cable in the image coordinate, and restoring the displacement into 3D world coordinate. In phase 1, a camera projection matrix, which contains the information of the intrinsic camera parameters and the camera pose, is estimated by using images taken with various angles and distances. In phase 2, the position of the target in the cable is tracked in the 2D image coordinate using the KLT (Kanade-Lucas-Tomasi) tracker. Finally, in phase 3, the cable displacement in the world coordinate is restored by combining the results from phase 1 (i.e., camera projection matrix) and phase 2 (i.e., location of the target in 2D image coordinate).

## 2. System Development

**Figure 1:**
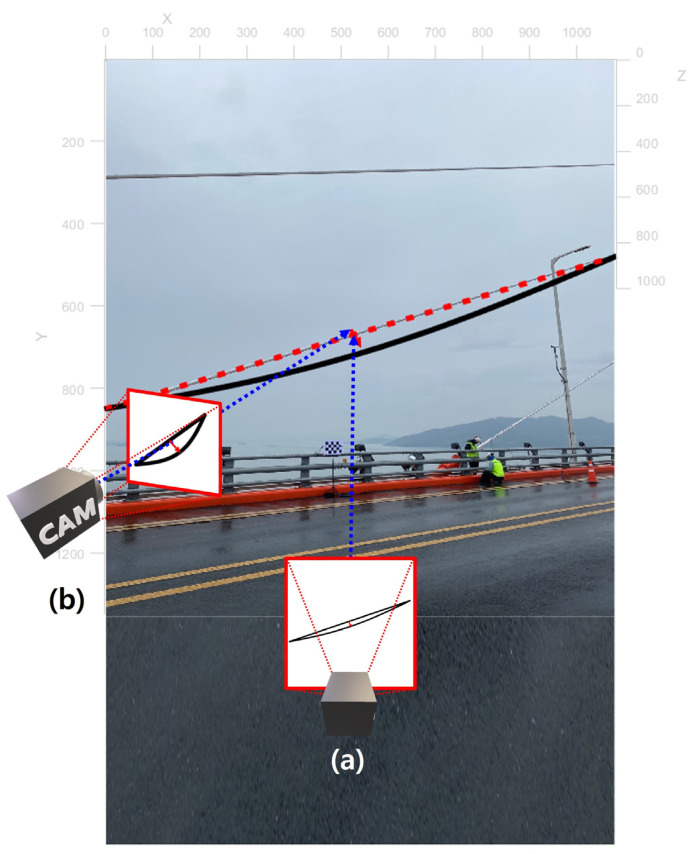
Camera locations for measuring cable displacement (**a**) front view and (**b**) side view.

The displacement estimated by using the conventional vision-based displacement measurement methods may contain large error depending on the camera location. When the cable displacement is parallel to the camera plane as shown in Figure 1a, the displacement of the structure can be accurately estimated by applying the conventional tracking algorithm. However, when using the vision-based displacement measurement method in the actual field, it is often difficult to install the camera perpendicular to the cable displacement. For example, when installing a camera at the opposite side of the bridge, the line of the sight might be blocked by an obstacle such as a vehicle. The line of sight of the camera can be secured when the camera is installed next to the cable as shown in Figure 1b. However, in this case, the displacement estimated using the side view camera will contain project error due to different field of view. Therefore, this study introduces a new method to estimate the displacement of the cable using the side view camera, even when the displacement of the cable-stayed bridge does not coincide with the camera plane.

**Figure 2:**
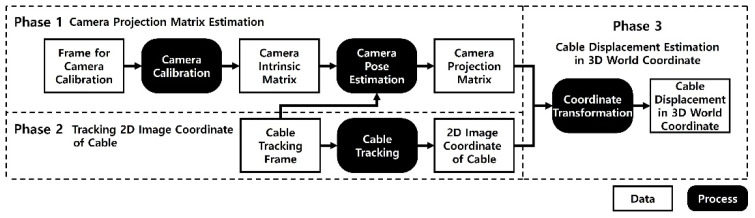
Overview for cable displacement measurement using side view video.

The proposed method for measuring the cable displacement using a side view camera is consist of three phases, as shown in Figure 2. First, a camera calibration process is conducted in phase 1 to remove radial distortion and to obtain camera projection matrix which contains the camera intrinsic parameters and the pose of the camera. Next, in phase 2, the feature points in the cables are tracked in the image coordinate. Finally, in phase 3, the displacement of the cable in the world coordinate can be calculated by combining the information from phase 1 (i.e., camera projection matrix) and phase 2 (i.e., tracking result in 2D image coordinate).

### 2.1. Phase 1. Camera Projection Matrix Estimation

The first step for measuring the cable displacement using side view camera is calibrating the camera. There are different purposes for conducting camera calibration in this study. First purpose is to remove a radial distortion in the images. Most of the commercial camera use wide-angle lens which has a large radial distortion. The camera calibration process can remove these distortions and minimize the error. Another reason for conducting camera calibration is to obtain camera projection matrix which contains intrinsic parameters such as focal length and the extrinsic parameters such as camera pose. A camera projection matrix is a matrix that projects the 3D coordinate points into the 2D image coordinate of the corresponding camera. In this study, the camera projection matrix is used to restore the displacement of the cable from the recorded video taken from the side.

**Figure 3:**
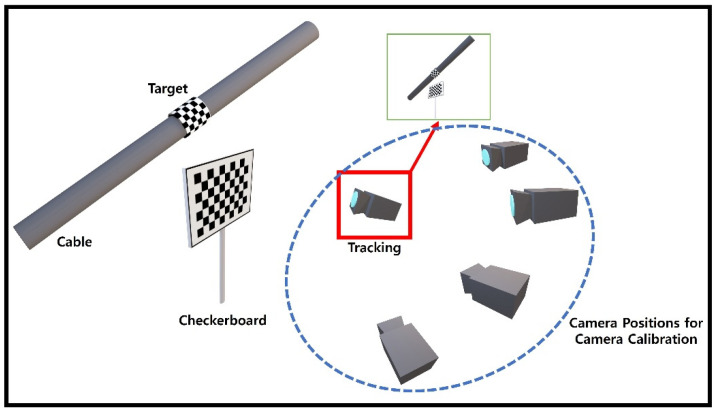
Configuration of Camera Calibration.

The configuration for the camera calibration process proposed in this study is shown in Figure 3. The camera projection matrix can be estimated by using multiple images taken from the Checkerboard at various locations and orientations. This study adopted the camera calibration method introduced by Zhang et al. [23].

As a result of camera calibration, camera intrinsic matrix and camera extrinsic matrix can be obtained. By combining camera intrinsic matrix K and camera extrinsic matrix [**R**|**t**]^T^, the camera projection matrix can be obtained as shown in Equation (1).

(1) $$\mathrm{Camera}\;\mathrm{Projection}\;\mathrm{Matrix}=\left[\begin{array}{c} R\\ t \end{array}\right]K=\left[\begin{array}{c} \begin{array}{ccc} C_{11} & C_{12} & C_{13} \end{array}\\ \begin{array}{ccc} C_{21} & C_{22} & C_{23} \end{array}\\ \begin{array}{ccc} C_{31} & C_{32} & C_{33} \end{array}\\ \begin{array}{ccc} C_{41} & C_{42} & C_{43} \end{array} \end{array}\right]$$

### 2.2. Phase 2. Cable Tracking in the Image Coordinate

**Figure 4:**
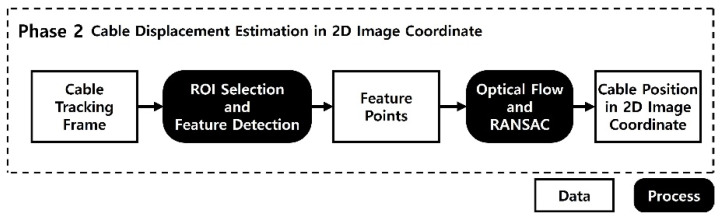
Overview of cable tracking in the image coordinate.

Phase 2 tracks the cable in the 2D image coordinate using the video taken from side view, as shown in Figure 4. The proposed method adopted the structural displacement measurement method proposed by Yoon et al. (2016), which used the optical flow based KLT tracker.

**Figure 5:**
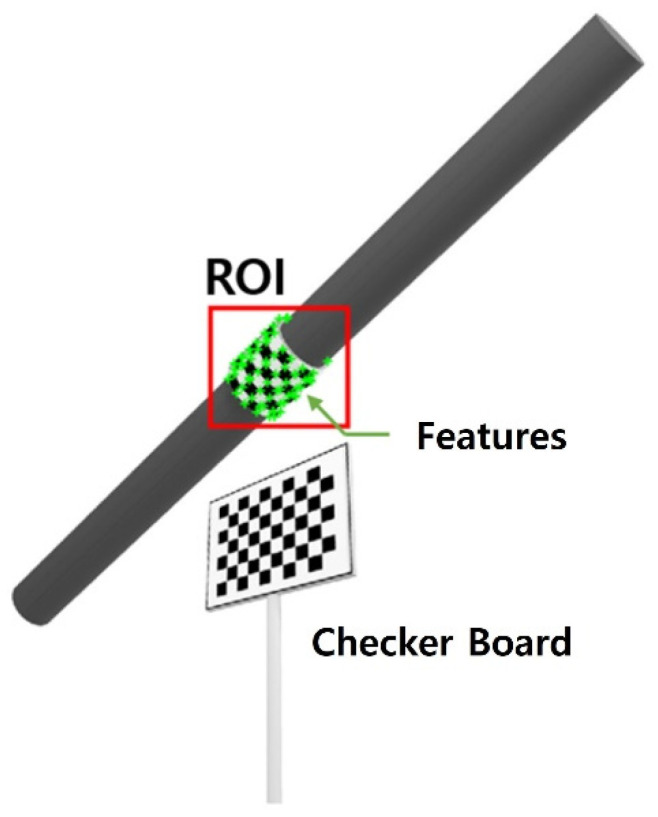
ROI selection and feature extraction.

The first step for tracking is to select a region of interest (ROI). The ROI should include as many as points in the cable, but avoid the points outside of the cable, as shown in Figure 5. If the ROI area is selected too large, feature points not related to the cable can be tracked. If the ROI area is selected too small, the number of feature points to be used for tracking may be insufficient. Therefore, it is important to select the ROI appropriately so that the features can be tracked in the further steps.

The next step is to extract feature points in the ROI. This study adopted Harris Corner points [24] as feature points. Harris Corner Detection is a method used to define a specific window (w) in an image and detect a part with a large difference in intensity within the window as a corner point while moving the window. Since the KLT tracker calculates the intensity difference between the previous frame and the next frame, a corner point is generally used as a feature point.

When a window in the image is moved by ∆*x*, ∆*y*, the difference in intensity within the window is calculated in the form of a sum of squares as in Equation (2).

(2) $$E\left(\Delta x,\Delta y\right)=\underset{w}{\sum }{\left[I\left(x_{i}+\Delta x,y_{i}+\Delta y\right)-I\left(x_{i},y_{i}\right)\right]}^{2}$$

In Equation (2), when the movement of the window (Δx,Δy) is very small, it can be summarized as in Equation (3).

(3) $$E\left(\Delta x,\Delta y\right)=\left[\Delta x\;\Delta y\right]M\left[\begin{array}{c} \Delta x\\ \Delta y \end{array}\right]=\left[\begin{array}{cc} \underset{w}{\sum }I_{x}{\left(x_{i},y_{i}\right)}^{2} & \underset{w}{\sum }I_{x}\left(x_{i},y_{i}\right)I_{y}\left(x_{i},y_{i}\right)\\ \underset{w}{\sum }I_{x}\left(x_{i},y_{i}\right)I_{y}\left(x_{i},y_{i}\right) & \underset{w}{\sum }I_{y}{\left(x_{i},y_{i}\right)}^{2} \end{array}\right]$$

Corner points can be select for the points that have large eigenvalues of **M** ($\lambda_{1}$, $\lambda_{2}$) obtained in the Equation (3). Since the eigenvalue analysis requires a large amount of computation, the following Equation (4) was used for detecting corner points.

(4) $$R=\det\left(M\right)-k*Tr{\left(M\right)}^{2}$$

Once the feature points are extracted, the next step is to track the feature points using an optical flow. If the intensity of the current frame is ***J***(x) and the intensity of the previous frame is ***I***(x), it can be expressed as in Equation (5).

(5) J(x)=I(x−d)=I(x)−g·d

In Equation (5), d is a displacement vector between consecutive frames, and $g\left(=\frac{\partial I}{\partial x},\frac{\partial I}{\partial y}\right)$ is a gradient vector expressed by the Taylor series, assuming that d is very small. The residue ϵ of the window based on the feature points is defined as in Equation (6).

(6) $$\epsilon =\int^{\;}{\left[I\left(v-d_{v}\right)-J\left(v\right)\right]}^{2}wdA=\int^{\;}{\left[I\left(x\right)-\frac{\partial I}{\partial v}\cdot d_{v}-J\left(v\right)\right]}^{2}wd=\int^{\;}{\left(h-\frac{\partial I}{\partial v}\cdot d_{v}\right)}^{2}wdA$$

In Equation (6), *w* is a weighting function and *h* is *I*(*x*) − *J*(*x*), which is the intensity difference of successive frames. In order to minimize residual, if ϵ is differentiated by d and the result is equal to 0, it can be expressed as Equation (7).

(7) $$\frac{d\epsilon }{dd}=\int^{\;}\left(h-g\cdot d\right)gwdA=0$$

As a result, the displacement vector d can be expressed as Equation (8).

(8) $$d=\frac{e}{G}$$

In Equation (8), $G=\int^{\;}gg^{T}wdA$, and $e=\int^{\;}\left(I-J\right)gwdA$. By applying this process to every frame, the displacement vector d for the feature point can be obtained.

Finally, the feature points of the cable in the image coordinates can be obtained by removing the outliers. The movement of the feature points of the current frame to the feature points of the next frame can be represented by a transformation matrix (T). In this study, the MLESAC (Maximum Likelihood Estimation SAmple Consensus) algorithm proposed by P.H.S. et al. [25] was used to estimate the transformation matrix (T) while removing the outliers.

### 2.3. Phase 3. Cable Displacement Estimation in 3D World Coordinate

Phase 3 estimates the displacement of the cable in the world coordinate using the camera project matrix obtained from Phase 1 and the feature points of the cable in the image coordinate obtained from Phase 2.

The relationship between a point in 3D world coordinate [X Y Z] and the projected point to an image [x y] is shown in the equation below.

(9) $$s\left[x\;y\;1\right]=\left[X\;Y\;Z\;1\right]\left[\begin{array}{c} \begin{array}{ccc} C_{11} & C_{12} & C_{13} \end{array}\\ \begin{array}{ccc} C_{21} & C_{22} & C_{23} \end{array}\\ \begin{array}{ccc} C_{31} & C_{32} & C_{33} \end{array}\\ \begin{array}{ccc} C_{41} & C_{42} & C_{43} \end{array} \end{array}\right]$$

where s is an arbitrary scale factor, $C_{ij}$ are the elements of the camera projection matrix. The camera projection matrix can be obtained from Phase 1, and the feature points in the 2D image coordinates cab be obtained from Phase 2. The unknown parameters in the Equation (9) are the scale factor and the point in the world coordinate. There are four unknowns (X, Y, Z, s) and three equations, and therefore the point in the world coordinate (X, Y, Z) cannot be directly solved from this equation.

Therefore, in this study, it was assumed that the out-of-plane displacement of the cable is negligible. By defining the out-of-plane direction to be the **Z**-axis, Z can be assumed to be a constant value, and the equation can be rewritten as below.

(10) $$\left[\begin{array}{ccc} s & X & Y \end{array}\right]=Z[C_{31}+C_{41}/Z\;C_{32}+C_{13}/Z\;C_{33}+C_{43}/Z]*{\left[\begin{array}{c} \begin{array}{ccc} x & y & 1 \end{array}\\ \begin{array}{ccc} -C_{11} & -C_{12} & C_{13} \end{array}\\ \begin{array}{ccc} -C_{21} & -C_{13} & C_{23} \end{array} \end{array}\right]}^{-1}$$

To simplify the Equation (10), the plane of the cable can be defined as where Z = 0. In this case, Equation (10) can be simplified as Equation (11), and the feature points in the world coordinate can be estimated from the feature points in the image coordinate.

(11) $$\left[\begin{array}{ccc} s & X & Y \end{array}\right]=\left[C_{41}+C_{13}+C_{43}\right]*{\left[\begin{array}{c} \begin{array}{ccc} x & y & 1 \end{array}\\ \begin{array}{ccc} -C_{11} & -C_{12} & C_{13} \end{array}\\ \begin{array}{ccc} -C_{21} & -C_{13} & C_{23} \end{array} \end{array}\right]}^{-1}$$

## 3. Validation Test

Overall, three validation tests were conducted to verify the performance of the proposed method. First, a simulation-based validation test was conducted to seek the accuracy of the proposed method in idealized condition. Next, a lab-scale validation test was conducted to validate the performance of the proposed method in the physical environment. Finally, an on-site validation test was conducted at a cable stayed bridge to seek the applicability of the proposed method to a real bridge. To analyze the performance of the proposed method in the validation tests, the displacements measured by proposed method were compared with the displacements measured by traditional KLT tracker (without compensating the effect of the side view). While the displacement measured by the proposed method is measured by a physical unit (i.e., mm), the displacement measured by the traditional KLT tracker is in pixel form. Therefore, to convert the pixel displacement into a physical unit, the scale factor for the traditional KLT tracker was obtained manually by measuring the length of a known object.

### 3.1. Simulation-Based Validation Test

**Figure 6:**
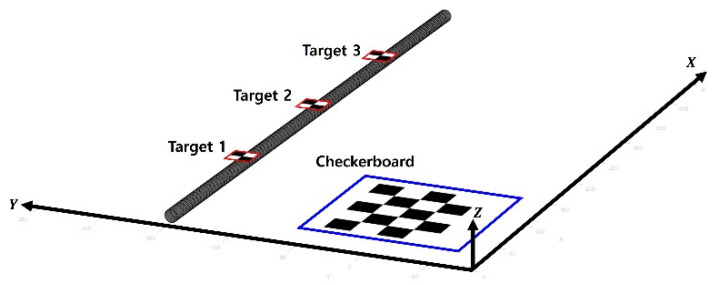
Configuration of the simulation-based validation test.

A simulation-based validation test was conducted to calculate the accuracy of the proposed method in idealized condition. A simulation for cable vibration was generated using MATLAB and Simulink. The simulation was then visualized and converted into a video with resolution of 1920 × 962 with 60 fps as if the video was taken from a distance of 2.6 m with side view camera. Overall, three targets were attached to the cable, and a 100 mm × 60 mm checkerboard were located next to the cable, as shown in Figure 6, defining the out-of-plane of the cable to the *Z*-axis.

**Figure 7:**
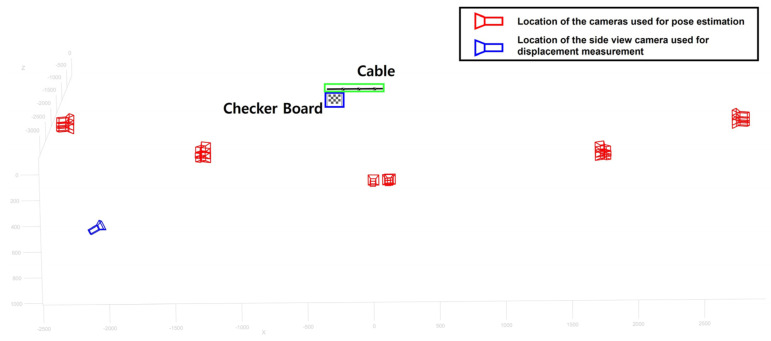
Camera pose estimation result for the simulation validation test.

The position (rotation and translation) of the camera estimated using the camera calibration is shown in Figure 7. Since the simulation test was performed in an ideal environment, the reprojection error was 0.02081. As a result, the intrinsic matrix was calculated as [8939.9, 0, 0; 0, 8940.4, 0; 680.5985, 796.9211, 1], and the rotation matrix and translation vector for the side view camera were obtained as [0.4329, −0.3142, 0.8449; 0.9013, 0.1369, −0.4110; 0.0135, 0.9394, 0.3425] and [123.2153, −13.6199, 2636.6], respectively. Finally, by combining the intrinsic matrix and the extrinsic matrix, the camera projection matrix was calculated as [4445.5, −2136.0, 0.8449; 7778.1, 896.2860, −0.4110; 353.6296, 8671.8, 0.3425; 2895,613.3 1978,938.5, 2636.0].

**Figure 8:**
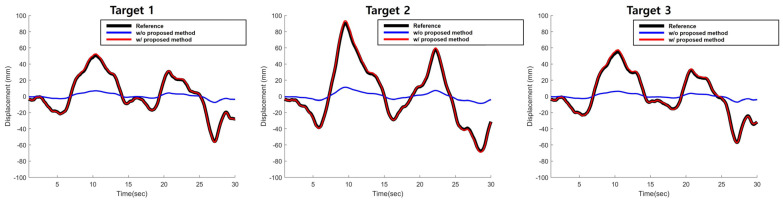
Estimated displacement for the simulation-based validation test.

Once the camera projection matrix was obtained, the displacement of the side view video was calibrated using the proposed method. Figure 8 illustrates the displacement results of the simulation validation test. To evaluate the performance of the proposed method, the calibrated displacement using the proposed method (red line) was compared with the reference displacement (black line) and the displacement without proposed method (blue line). As the figure indicates, the proposed method was able to estimate the displacement even with the side view, while the without proposed method) showed a significant difference with the reference displacement.

The RMSE (root mean square error) of the displacement is shown in Table 1. The proposed method showed RMSE of 0.7001 mm, 1.2789 mm and 0.9579 mm, respectively, for each target, while the RMSE of the displacement without proposed method were of 20.6601 mm, 32.6588 mm and 22.6273 mm. The proposed method could reduce the average RMSE from 25.3154 mm to 0.9790 mm.

**Figure 9:**
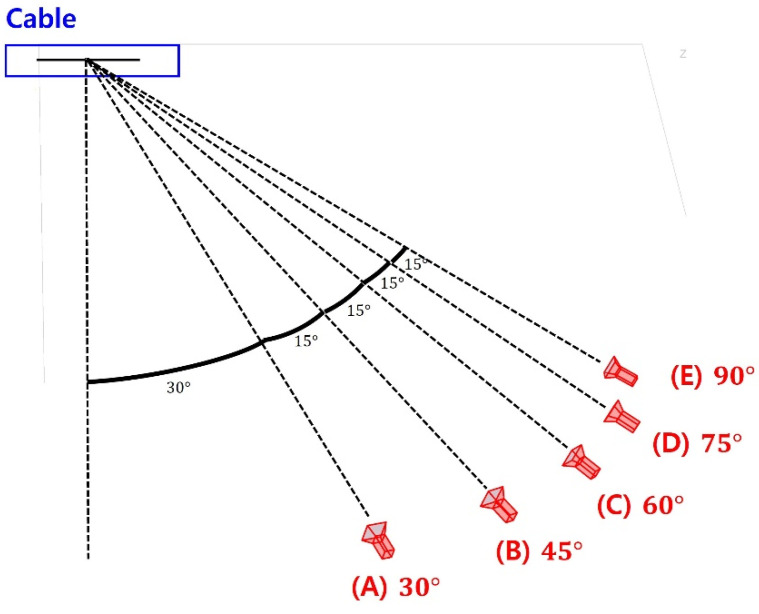
Location of the camera with various angles.

**Figure 10:**
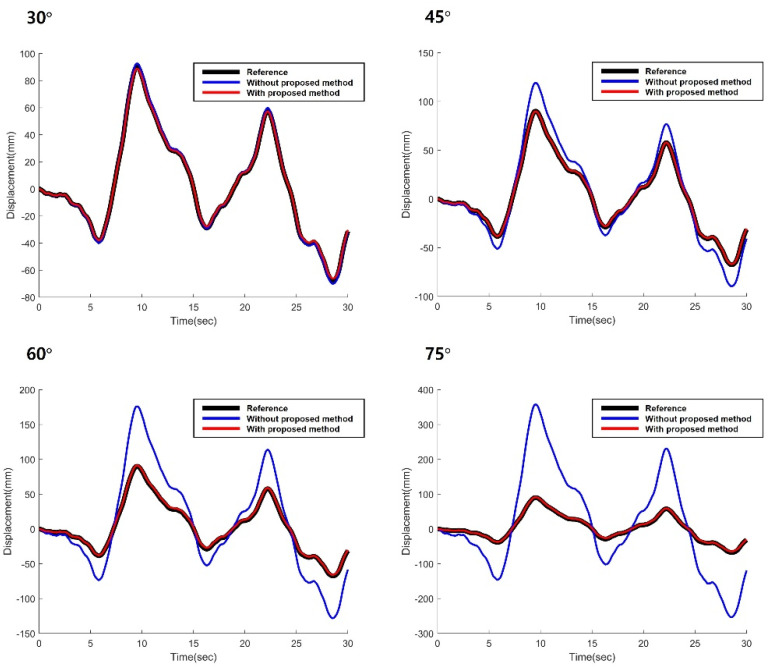
Estimated cable displacement with various angles.

To quantitatively analyze how much the angle affects the performance of the proposed method, the simulation-based validation test was repeated by changing the angle between the plane of the camera and the plane of the cable displacement from 30° to 90°. The camera was assumed to be installed at 3 m away from the cable, as shown in Figure 9. The estimated displacement using the proposed method and the traditional KLT tracker (without compensating the side angle effect), compared to the reference displacement, which is shown in Figure 10, and the RMSE, which is shown in Table 2. As shown in the figure, the displacement estimated using the proposed method and the displacement using the traditional KLT were almost identical to the reference displacement when the camera was installed at an ideal location (no side angle). When the angle between the plane of the camera and the plane of the cable displacement was 30°, the RMSE for the traditional KLT was 1.5135 while the RMSE for the proposed method was 0.4356. When the angle increased, the RMSE for the traditional KLT significantly increased while that of the proposed method increased slightly. When the angle reached to 80°, both the proposed method and the traditional KLT tracker were not able to estimate the cable displacement because the feature points in the cable were not visible.

From the simulated validation test, it has been proven that the proposed method could reduce the projection error due to the side angle view, especially when the angle between the camera plane and the displacement plane is around 75°. However, we could also find the limitation of the proposed method; the proposed method cannot compensate the side angle effect, if the angle is equal or larger than 80°.

### 3.2. Lab-Scale Validation Test

**Figure 11:**
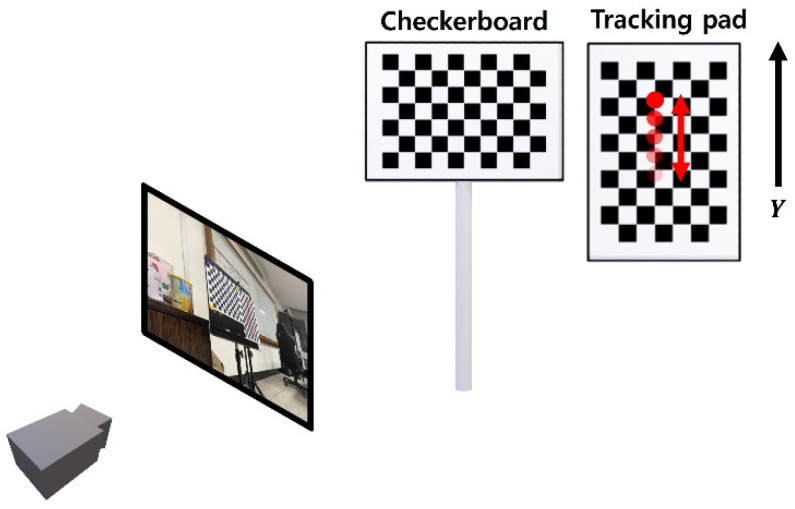
Configuration of the lab-scale validation test.

A lab-scale validation test was conducted to validate the performance of the proposed method in physical environment as shown in Figure 11. To obtain the reference displacement accurately, the lab-scale validation test was conducted by tracking a point in a tracking pad. Points in the tracking pad was assumed to moved only in the *Y*-axis direction. The video was recorded by a side view camera with 4032 × 3024 resolution and 30 fps which was installed about 70 cm away from the checkboard.

**Figure 12:**
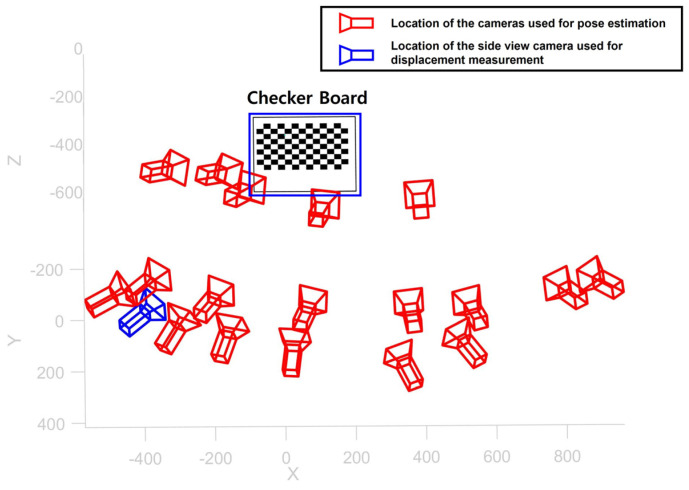
Camera pose estimation result for the lab-scale validation test.

As a first step, pose of the camera was estimated by taking total of 19 photos from different positions, as shown in Figure 12. The reprojection error for camera calibration was 0.0679 mm, which was slightly larger than that of simulation test. As a result, the intrinsic matrix was calculated as [3018.2, 0, 0; 0, 3031.1, 0; 2020.8, 1421.6, 1], and the rotation matrix and translation vector for the side view camera were obtained as [0.6830, 0.0358, 0.7295; 0.1388, 0.9742, −0.1777; −0.7171, 0.2226, 0.6605] and [−100.4591, −118.3787, 686.9710], respectively. Finally, by combining the intrinsic matrix and the extrinsic matrix, the camera projection matrix was calculated as [3535.7, 1145.5, 0.8295; 59.7363, 2700.4, −0.17773; −829.53, 1613.8, 0.6605; 1085,047.0, 617,772.4, 686.97].

Once all of the distortions of the images were removed and camera projection matrix was obtained, the feature points in the cable in the image coordinate were measured. In this lab-scale validation test, the points in the 2D image coordinates were obtained using the checkerboard points detection method proposed by A Geiger et. al. [26]. It was assumed that each point moved one space (20 mm) along the *Y*-axis per each time step. Finally, the points in the image coordinate were converted into the world coordinate using proposed method.

**Figure 13:**
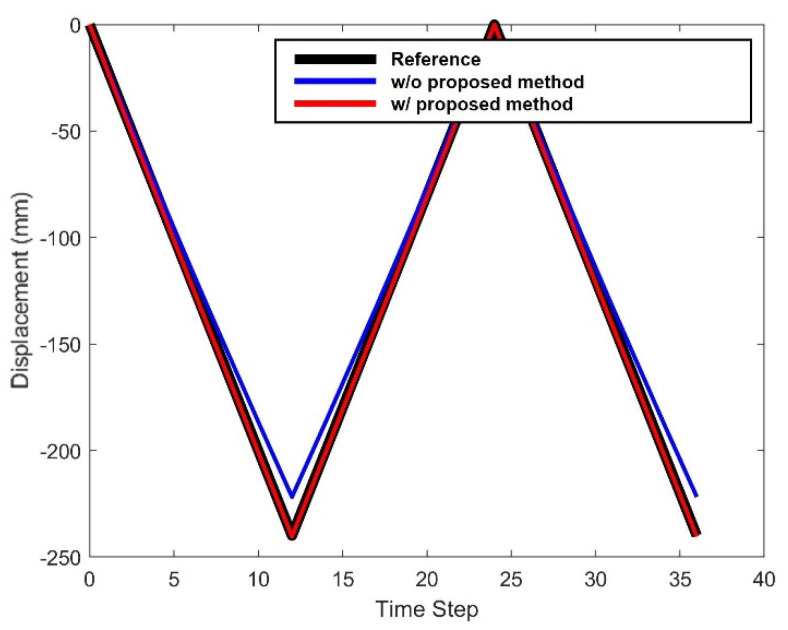
Estimated displacement for the lab-scale validation test.

Figure 13 shows the results of the displacement without (blue) and with (red) applying the proposed method. The error for both methods were lower than the error of the simulation-based validation test, since the camera was installed at a closer distance and the tracking error was negligible. The RMSE of the displacement without the proposed method was 9.0292 mm, and the RMSE of the displacement with the proposed method was 0.9318 mm. From the result, it can be concluded that the proposed method can significantly reduce the error of the displacement by compensating the projection error of the side view video.

### 3.3. On-Site Validation Test

**Figure 14:**
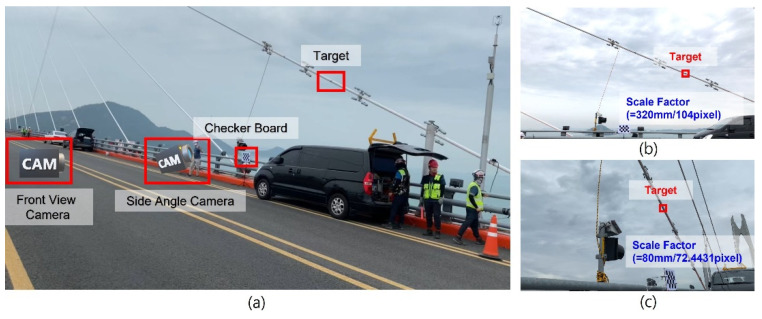
(**a**) Configuration of the on-site validation test with an image taken from (**b**) front view camera and (**c**) side view camera.

On-site validation test was conducted at Cheonsa Bridge, South Korea to seek the applicability of the proposed method. The configuration of the on-site validation test is shown in Figure 14a. A target was attached to the cable to be measured, and a checkerboard was installed next to the cable. The axis of the global coordinate system X, Y, Z was defined according to the checkerboard. Two cameras with resolution of 3840 × 2160 and 30 fps were installed at the site, one camera with a front view (Figure 14b), and another camera with side view (Figure 14c). The front view camera was installed approximately 15 m away from the checkerboard, and the side view camera was installed 3 m away from the checkerboard.

**Figure 15:**
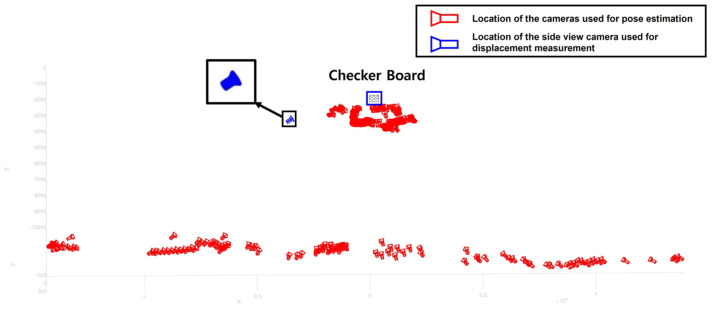
Camera pose estimation result for the on-site validation test.

To estimate the pose of the side view camera, a total of 237 images were taken from various locations with different angles as shown in Figure 15. The reprojection error for the camera calibration was 0.3563 pixels, which was higher compared to the simulation and the lab-scale test. As a result of, the intrinsic matrix was estimated as [3102.2, 0, 0; 0, 3081, 8, 0; 1.938.8, 1043.8, 0], and the extrinsic matrix of the side view camera was estimated as [0.4706, 0.2781, 0.8374; 0.0671, 0.9350, −0.3482; −0.8798, 0.2201, 0.4214]. By combining the intrinsic matrix and the extrinsic matrix, the camera projection matrix was calculated as [3083.4, 1731.1, 0.8374; −467.0903, 2518.0, −0.3482; 8818,250.5, 6901,126.8, 3654.1].

Next, the ROI was selected from the initial frame of the side view video and the feature points in the image coordinate were tracked for each video frame. Then, the feature points in the image coordinate were transformed into the world coordinate using the camera projection matrix. Finally, the displacement of the cable in the world coordinates were calculated.

In this on-site validation test, a displacement measured by using a front view camera were used as the reference displacement. To obtain the reference displacement, the following procedure was conducted. A video was recorded by a front view camera as shown in Figure 14b. Feature points were tracked in 2D image without applying the proposed method. Next, the scale factor was calculated using the known length in the image (i.e., checkerboard). Finally, the displacement was calculated. While the front view camera was used as a reference displacement, it is not a perfect measurement since it will contain a tracking error and a projection error.

**Figure 16:**
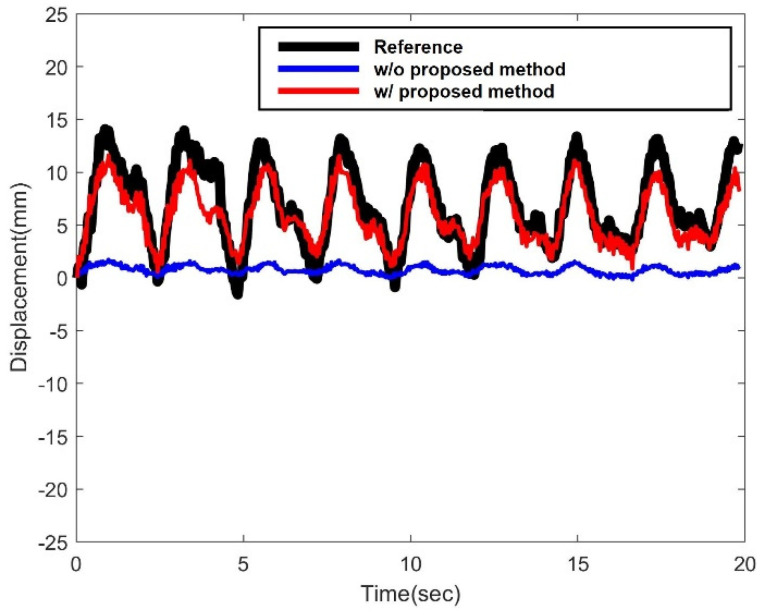
Estimated displacement for the on-site validation test.

Figure 16 shows the displacement of the cable without (blue) and with (red) the proposed method, together with the front view camera result (black). The RMSE of the proposed method was 1.6803 mm, while the RMSE of the conventional method showed 6.4672 mm. The proposed method estimated the cable displacement more accurately compared to the without proposed method by 4.7869 mm. Since the reference displacement obtained from the front view camera contains the error, the actual RMSE are not accurate; the actual RMSE might be lower than what we achieved. However, it was shown clearly that the proposed method was able to reduce the error significantly when measuring a displacement from a side view video.

## 4. Conclusions

This paper presented a new method for estimating the displacement of a cable from a side view camera. The proposed method was comprised of three phases. Phase 1 estimates the camera projection matrix which contains the intrinsic matrix and the information related to the pose of the camera. Phase 2 tracks the feature points of the cable in the image coordinate. Finally, in Phase 3, the cable displacement in the world coordinate is restored by combining the result of phases 1 and 2.

Through the simulation-based validation test, it was possible to evaluate the accuracy of the proposed method under ideal conditions. In addition, by conducting the lab-scale validation test, it was possible to confirm the performance of the proposed method in the physical environment. Finally, an on-site validation test was conducted at Cheonsa Bridge, South Korea, to seek the applicability of the proposed method to the real cable-stayed bridge. Compared to the results of the simulation-based validation test and the lab-scale validation test, the error occurred larger in the on-site validation test. This was due to various environmental conditions, but also due to the inaccuracy of the reference value. However, even taking that into account, it was possible to show that the proposed method can significantly reduce the error compared to the traditional method without proposed method. Therefore, it is expected that the proposed method will contribute toward vision-based cable displacement measurement by broadening the camera installation locations.

## Figures and Tables

**Table 1:** RMSE of the estimated displacement with and without proposed method.

RMSE (mm)	w/ Proposed Method	w/o Proposed Method
Target 1	0.7001	20.6601
Target 2	1.2789	32.6588
Target 3	0.9579	22.6273
**Average**	**0.9790**	**25.3154**

**Table 2:** RMSE of the estimated displacement with various angles.

Angle	w/Proposed Method	w/o Proposed Method
30°	0.4356	1.5135
45°	0.4367	9.6278
60°	1.1652	27.4040
75°	1.6531	86.9786
80°	N/A	N/A
90°	N/A	N/A
